# A pilot study to profile salivary angiogenic factors to detect head and neck cancers

**DOI:** 10.1186/s12885-018-4656-3

**Published:** 2018-07-13

**Authors:** L. van der Merwe, Y. Wan, H. J. Cheong, C. Perry, C. Punyadeera

**Affiliations:** 1 The School of Biomedical Sciences, Institute of Health and Biomedical Innovations, Queensland University of Technology, 60 Musk Avenue, GPO Box 2434, Kelvin Grove, Brisbane, QLD 4059 Australia; 2 The School of Chemistry & Molecular Biosciences, The University of Queensland, Brisbane, Australia; 3 Department of Otolaryngology, Princess Alexandra Hospital, 199 Ipswich Road, Woolloongabba, Brisbane, QLD 4102 Australia; 4 Translational Research Institute, Woolloongabba, Brisbane, QLD 4102 Australia

**Keywords:** Angiogenesis, Saliva, Human papillomavirus, Head and neck squamous cell carcinoma

## Abstract

**Background:**

Early diagnosis of head and neck squamous cell carcinoma (HNSCCs) is an appealing way to increase survival rates in these patients as well as to improve quality of life post-surgery. Angiogenesis is a hallmark of tumor initiation and progression. We have investigated a panel of angiogenic factors in saliva samples collected from HNSCC patients and controls using the Bio-Plex ProT^M^ assays.

**Methods:**

We have identified a panel of five angiogenic proteins (sEGFR, HGF, sHER2, sIL-6Ra and PECAM-1) to be elevated in the saliva samples collected from HNSCC patients (*n* = 58) compared to a control cohort (*n* = 8 smokers and *n* = 30 non-smokers).

**Results:**

High positive correlations were observed between the following sets of salivary proteins; sEGFR:sHER2, sEGFR:HGF, sEGFR:sIL-6Rα, sHER2:HGF and sHER2:sIL6Ra. A moderate positive correlation was seen between FGF-basic and sEGFR.

**Conclusion:**

We have shown that angiogenic factor levels in saliva can be used as a potential diagnostic biomarker panel in HNSCC.

**Electronic supplementary material:**

The online version of this article (10.1186/s12885-018-4656-3) contains supplementary material, which is available to authorized users.

## Background

Head and neck squamous cell carcinoma (HNSCC) patients are diagnosed at an advanced stage due to the lack of early diagnostic methods, as such approximately 50% of patients die within 5 years of diagnosis [1–7]. The majority of HNSCC patients at diagnosis present with tumours that are often large and may have developed regional lymph node metastases or distant metastases. The survival rates and the quality of life in HNSCC patients are directly associated with the size of primary tumour at diagnosis. Major etiological risk factors for HNSCC include the synergistic effects of tobacco use and excessive alcohol consumption [8, 9]. In addition, human papillomaviral infections (high risk subtypes HPV-16, − 18, − 31, − 35 etc. [10, 11], account for a subgroup (approximately 20–50%) of HNSCC that arise from the oropharynx and have distinct clinicopathological and biological features [6, 12, 13]. The current diagnostic strategies for these patients rely on histological analyses of tumour tissue samples followed by PET-CT scans, which have demonstrated to be inadequate, due to the high frequency of disease recurrences (2years from diagnosis) [1].

Most tumours exploit signals generated from cellular and non-cellular extracellular matrix (ECM) components to promote tumour growth and dissemination. The process of new blood vessel formation (angiogenesis) is initiated to facilitate tumours with the means to supply nutrients to accelerate their growth, as well providing avenues for eventual metastasis through the vascular system. Angiogenesis is a critical process, which is paramount to the progression and establishment of HNSCC tumours [14]. Pro- and anti-angiogenic factors are released from tumour cells and inflammatory associated cells [15]. As tumour mass increases, so does the demand for nutrients and oxygen, and in response to this need, tumour cells release pro-angiogenic signals expanding the tumour vascular network [14].

The role of saliva is being extensively researched both as a screening and a diagnostic tool in detecting oral and systemic diseases e.g. heart failure, cancer, ischemic heart disease, diabetes, rheumatoid factor diseases and other systemic diseases [16–21]. As such, salivary testing is a rapidly expanding field and may provide an inexpensive, easily accessible and non-invasive alternative to traditional tissue, blood or urine testing [22, 23]. The ease of conducting saliva-based tests makes it an attractive option for large population based screening studies, especially in children and in the elderly. Saliva is an ideal diagnostic medium for point of care platforms, for home based testing and ideal when there is a need for repeated sampling to monitor and manage the disease progression [24]. We hypothesise that a composite profiling of salivary angiogenic factors can discriminate healthy controls from HNSCC patients. This study aims to investigate whether salivary angiogenic factors can discriminate HNSCC patients from controls.

## Methods

### Study design

This study is approved by the University of Queensland (HREC no.: 2014000679) and Queensland University of Technology (HREC no.: 1400000617) Medical Ethical Institutional Boards and the Princess Alexandra Hospital’s (PAH) Ethics Review Board (HREC no.: HREC/12/QPAH/381). Written informed consents were received from all participants before sample collection.

Sample size power calculation was estimated from our previously published work [20]. In order to achieve an area under the curve (AUC) of 0.80 with a power of 0.80 and type I error rate of 0.05, a minimal of *n* = 50 patients is needed to demonstrate the diagnostic value of discovered salivary angiogeneic factors. We have recruited controls consisting of both smokers (*n* = 8) and non-smokers (*n* = 30). Exclusion criteria for controls included the existence of cancer, periodontal diseases, autoimmune disorders, infectious diseases, malignant disease and recent trauma. All of the controls were of > 40 years of age. All controls were generally in good health, were not on any medication (oral contraceptive excluded) and practiced regular oral hygiene. HNSCC patient cohort consists of HPV-negative (*n* = 30) and HPV-positive patients (*n* = 28). Table 1 presents the demographic and clinical characteristics of our study cohort.

**Table 1:** A summary of demographics of HNSCC patients and healthy control cohorts

Parameter	Non-smoker control	Smoker control	HNSCC patient HPV-	HNSCC patient HPV+
Number (*n*)	8	30	30	28
Age: mean (range)	51 (42–63)	51 (40–74)	61 (42–74)	58 (46–72)
Gender (M:F)	6:11	10:5	17:6	20:0
Ethnicity				
Caucasian	16	15	21	20
Other	1	Nil	2	Nil
Smoke status				
Smoker	N/A	15	9	8
Ex-smoker	N/A	N/A	10	8
Non-smoker	17	N/A	3	4
Unspecified	N/A	N/A	1	Nil
Tumour Stage				
T0/T1/T2	N/A	N/A	14	8
T3/T4	N/A	N/A	7	11
Unspecified	N/A	N/A	2	1

### Saliva sample collection and processing

Volunteers were asked to refrain from eating and drinking for an hour prior to donating saliva samples. Saliva samples were collected based on our previous publications [25–27]. The volunteers were asked to sit in a comfortable position and were asked to rinse their mouths with water to remove food debris. They were then asked to pool saliva in their mouths and expectorate directly into a 50 mL Falcon tube kept on ice. Saliva samples were transported from the hospital to the laboratory on dry ice. Samples were stored at -80 °C until further analysis.

### The use of bio-Plex pro™ human Cancer biomarker panel with saliva samples

The Bio-Plex Pro™ Human Cancer Biomarker Panel 1, 16-plex (cat no. 171-AC-500 M; Kit lot number = 500,034,952 and 50,045,678) was used to investigate levels of known cancer proteins in saliva samples collected from controls as well as HNSCC patients. Bio-Plex is a multiplexing high throughput system, enabling the quantification of up to 100 different analytes in a single sample [28]. The Bio-Plex Pro™ Human Cancer Biomarker Panel 1 interrogates a range of cellular functions including angiogenesis, metastasis, inflammation, cell adhesion, cell proliferation and apoptosis. Angiogenic factors included in this assay are related to HNSCC in three different ways.

The Bio-Plex Pro™ assays were run as per manufacturer’s guidelines. All standards, samples and controls were prepared in a sample diluent HB buffer (with addition of bovine serum albumin to 0.5% final). A total volume of 50 μL of 1:1 diluted samples were used per well. All standards, samples and controls were assayed in duplicate. Briefly, 50 μL of 1× antibodies coupled to magnetic beads were added to the 96 well plate, followed by washing the plate 3 times with 100 μL of Bio-plex wash buffer using Bio-Plex Pro™ II Wash Station (Bio-Rad Laboratories, Inc., Hercules, California, U.S.A.). The standards, samples and controls (50 μL) were then added to the plate. Plates were then incubated for 1 hour at room temperature (RT) with shaking at 850 rpm. The magnetic beads were then washed 3 times as described before. Then 25 μL of 1× detection antibody was added and incubated for a further 30 min at RT with shaking at 850 rpm. The magnetic beads were then washed 3 times as described before. Streptavidin-PE (1×, 50uL) was added to each well and incubated for 10 min at RT with shaking at 850 rpm. A final 3 x wash cycle was performed. The beads were resuspended in 125 μL assay buffer, shaken at 850 rpm for 30 s and read on Bio-Plex system. Bio-Plex xMAP technology encompassing a flow cytometer with dual laser was used to measure bound molecules on the beads. In addition, the high-speed digital signal processor was used to efficiently manage the data produced. Bio-Plex Manager™ Software was used to plot standard curves with logistic 5 PL regression.

### Statistical analysis

Statistical data analysis was performed using GraphPad Prism 6 software version 6.03 (GraphPad Software Inc., La Jolla, CA, USA) and R version 3.1.2 (R Development Core Team. Vienna, Austria). Bio-Plex manager software was used to generate standard curves and to extrapolate concentrations of the analytes. Prior to the statistical analysis, coefficient of variation (CV), percentage recovery and normality of the data was checked. CV for the assay was used to assess distribution of data for sample replicates. Acceptable CV is < 30%, samples with CVs above this range were eliminated or rerun. Two quality controls (QC) were run in parallel to the Bio-Plex assay. QCs are samples with known concentration of analyte prepared by the manufacturer. Percentage recovery of the QCs is used to the test accuracy of our assay. Percentage recovery between 70 to 130% is considered acceptable verifying the assay has an accurate interpretation of the samples assayed. Once the percentage CV and recovery was verified, statistical analysis of the results was performed following the guidelines below.

#### Shapiro-wilk normality test

Firstly Shapiro-wilk normality test was used to determine the normality of the data set. The null hypothesis is that the population is normally distributed. A *p*-value, *p* < 0.05 reject the null hypothesis and *p* > 0.05 were considered normally distributed.

#### Data log transformation

If the data set failed Shapiro-wilk normality test (*p* < 0.05), data was normalised by log transformation, *y* = Log(y), and normality of the data retested.

#### Multiple comparison tests

One-way ANOVA with post hoc test (Tukey test) was used for normal data whilst Kruskal-Wallis Test with post-hoc test (Tukey test) was used for data sets failing normality test. A *p* < 0.05 was considered to be significantly different.

#### Spearman’s rank correlation (nonparametric)

R package “corrgram” [29] was used to plot a correlation matrix between wo variables. Spearman’s correlation coefficient (*r*_*s*_*)* measures the strength of a monotonic relationship between paired data. The nearer *r*_*s*_ is to ±1, indicates are stronger monotonic relationship.

## Results

### Bioplex data

**Fig. 1:**
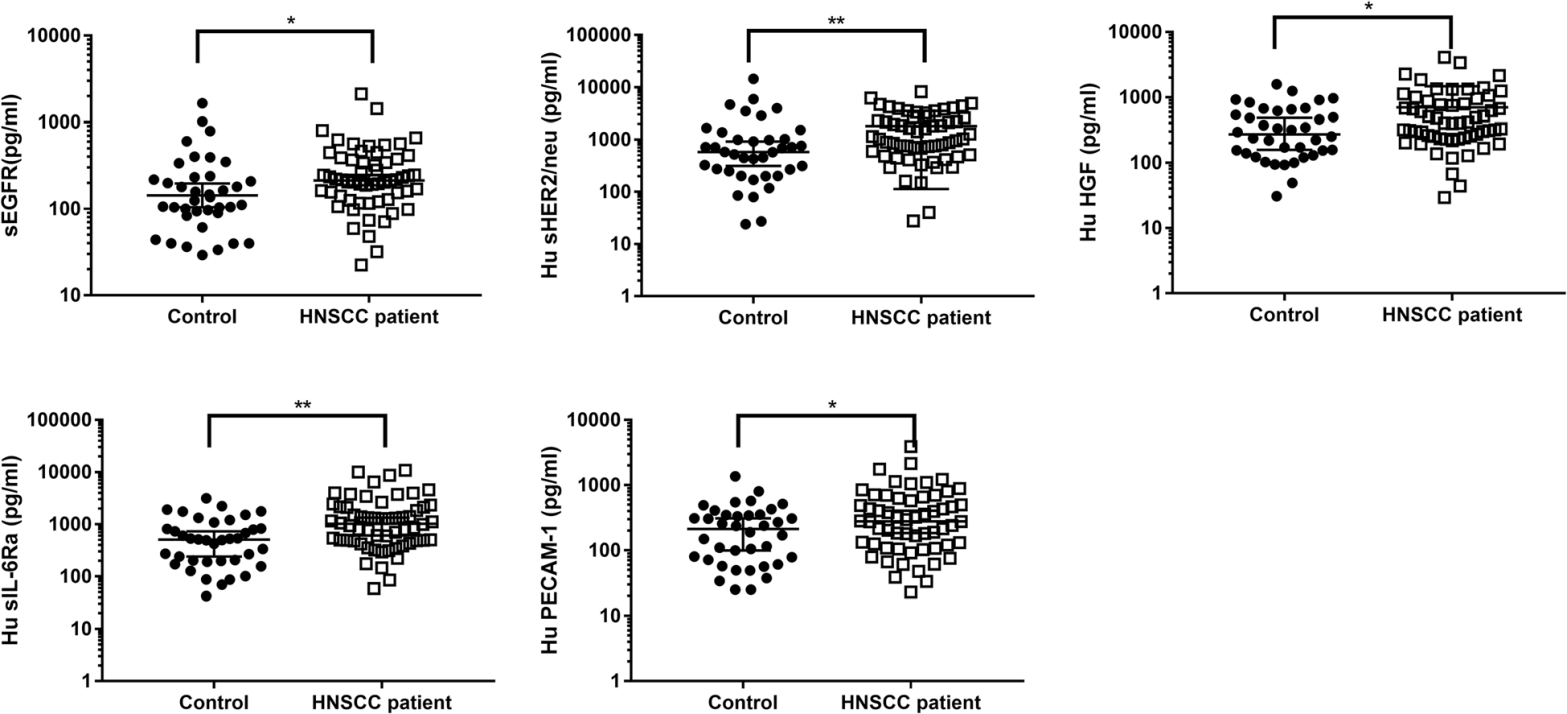
Five angiogenic factor concentrations in saliva samples collected from head and neck squamous cell carcinoma patients (*n* = 58) and healthy controls (*n* = 38). *p < 0.05 and **p < 0.01

**Fig. 2:**
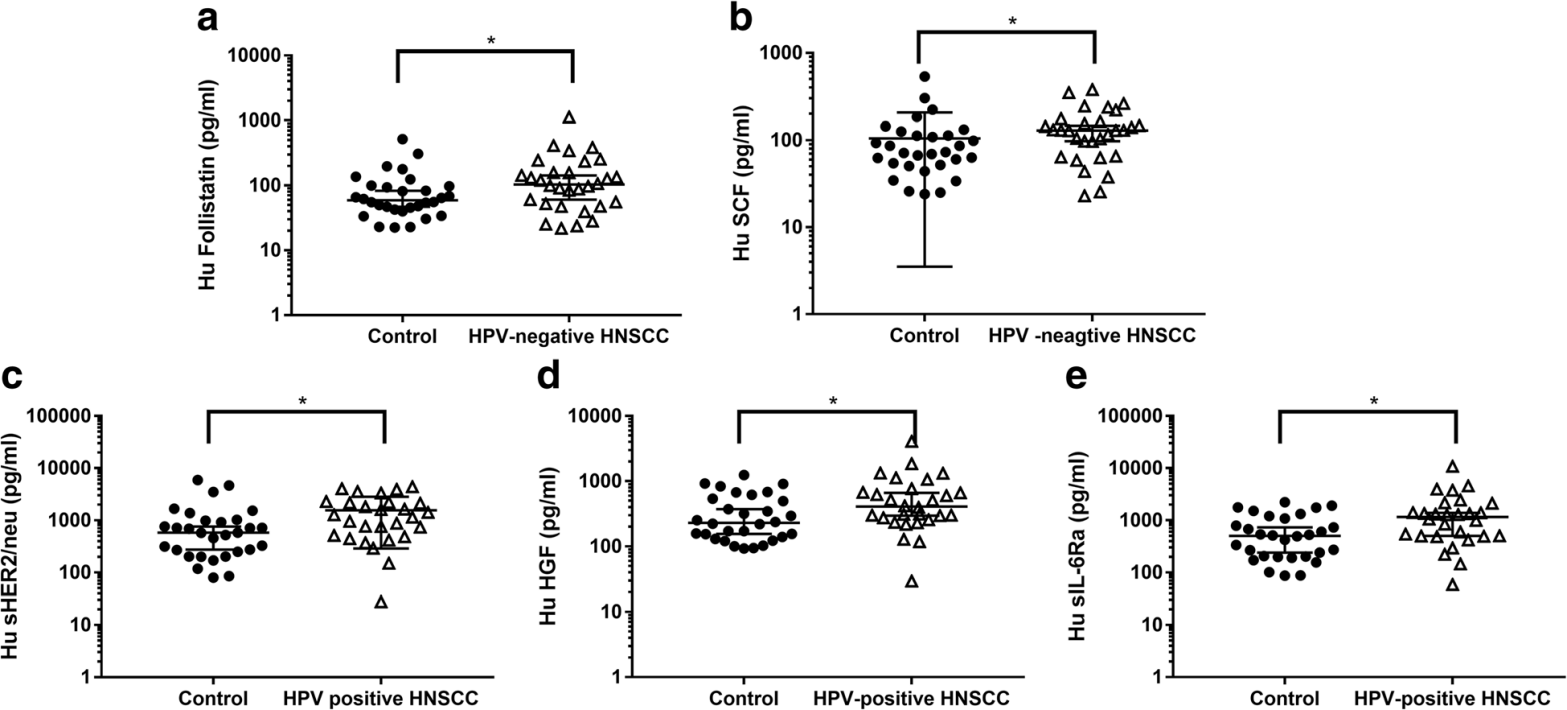
**a**, **b** Salivary angiogenic factor concentrations between HPV-negative HNSCC patients (*n* = 30) and healthy controls (*n* = 38) and (**c**, **d**, **e**) and angiogenic factor concentrations in the saliva collected from HPV-positive HNSCC patients (*n* = 28) and healthy controls. *p < 0.05 and **p < 0.01

The Bio-Plex Pro™ assay was used to quantify the concentrations of 16 angiogenic factors in saliva samples collected from HNSCC patients and healthy controls. There were no significant differences in the angiogenic factor concentrations (sEGFR, *p* = 0.6863; sIL-6Rα, *p* = 0.7123; HGF, *p* = 0.4075, sHER2, *p* = 0.6863, and PECAM-1 *p* = 0.3111) in saliva samples collected from non-smoker healthy controls and smoker healthy controls. This would mean that smoking has no influence on the angiogenic factors measured above. As such, the salivary data for smoker and non-smoker controls were combined as “controls”. Out of the 16 proteins investigated, five angiogenic factors (sEGFR, sHER2, HGF, sIL-6Ra and PECAM-1) were significantly different between saliva samples collected from controls and HNSCC patients (Fig. 1). Follistatin and SCF were found to be significantly different between the saliva samples collected from HPV-negative HNSCC patients and healthy controls (Fig. 2a and b). In contrast, sHER2/neu, HGF and sIL-6Ra levels were significantly elevated in saliva samples collected from HPV-positive HNSCC patients and healthy controls (Fig. 2c-e). FGF-basic, Follistatin, prolactin and SCF levels were found to be significantly different between saliva collected from HPV-negative patients and HPV-positive patients.

### A correlation matrix for salivary angiogenic factors

**Fig. 3:**
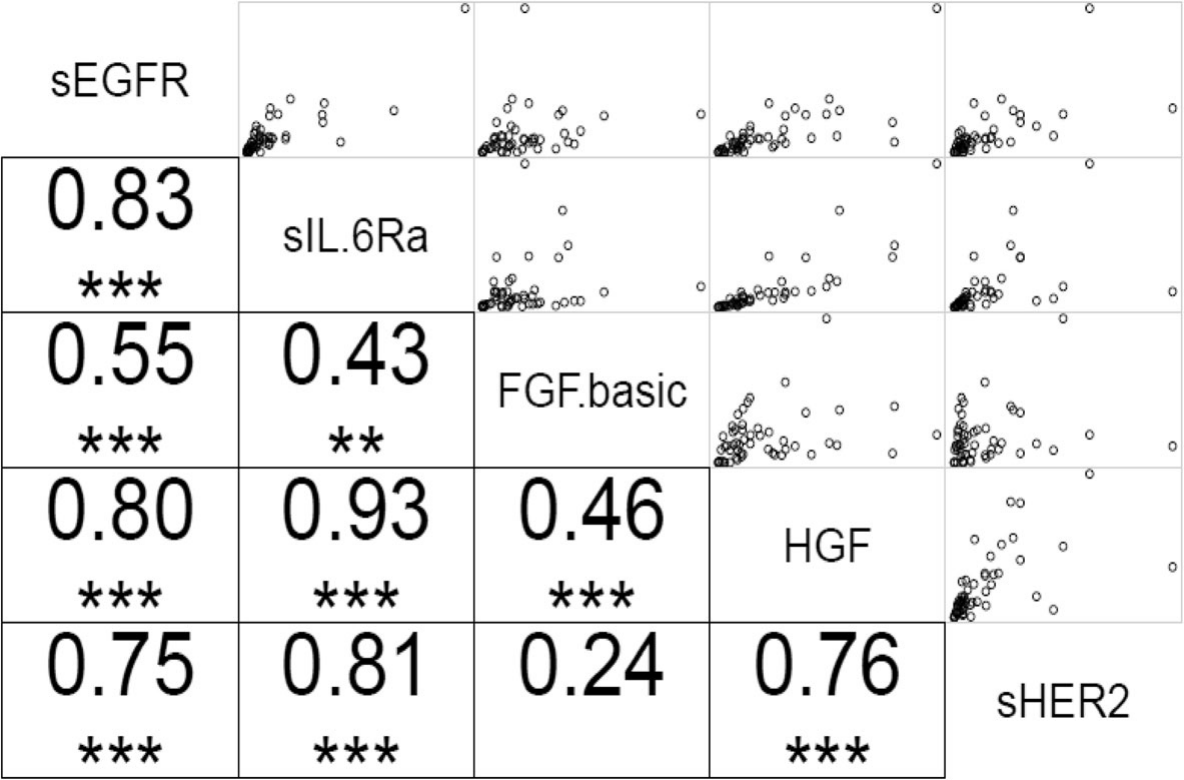
The correlation matrix for five salivary angiogenic factors

A Spearman’s correlation was performed to determine the strength of a monotonic relationship between salivary angiogenic factor concentrations. High positive correlations were observed between the following sets of salivary proteins; sEGFR:sHER2, sEGFR:HGF, sEGFR:sIL-6Rα, sHER2:HGF and sHER2:sIL6Ra. A moderate positive correlation was observed between FGF-basic and sEGFR (Fig. 3).

### Multivariate receiver operating characteristic curve generated using salivary angiogenic factors

**Fig. 4:**
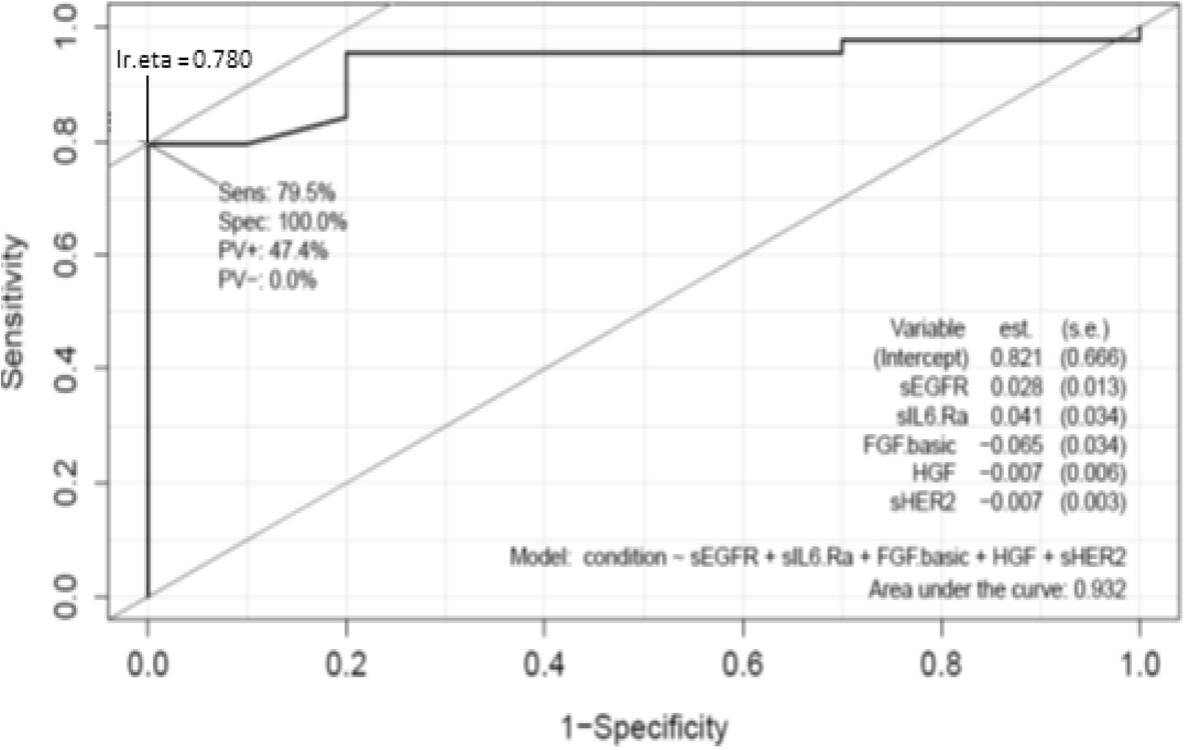
Performance of the panel in detecting controls vs head and neck cancer patients. Multivariate receiver-operating characteristics curve when all of the five salivary angiogenic factors are combined, comparing normal healthy controls (*n* = 38) with HNSCC patients (*n* = 58)

We evaluated the sensitivity and specificity of individual angiogeneic factors as well as combining them into a panel. Individual angiogenic factor diagnostic performance appear in the Additional file 1: Table S1. When combining all five of the angiogenic factors into a panel gave an AUC of 0.932; sensitivity of 79.5% and specificity of 100% (Fig. 4).

## Discussion

Despite major improvements in its management, over 350,000 people die annually worldwide from HNSCC, in comparison to other cancer types (breast, colorectal and prostate cancers). Approximately two-thirds of HNSCC patients are diagnosed at an advanced-stage of the disease (stage III to IVB), limiting the effectiveness of treatments, and hence reducing their chance of survival [30]. Metastases (both loco regional and distant) remains the major cause of death in HNC patients [31]. Angiogenesis plays an important role in tumour growth and metastasis. Regulation of the angiogenic process depends on the balance between the growth promoting factors and growth inhibitory factors. Numerous inducers of angiogenesis have been identified and known to play a role in tumour metastasis [32, 33]. Saliva testing, a non-invasive alternative to serum testing, has gained momentum in recent years. Saliva testing is inexpensive and easy to use and one can collect multiple samples simultaneously or sparsely from a patient. In this study, we have identified a panel of five angiogenic proteins that are elevated in the saliva samples collected from HNSCC patients compared to a control cohort with an AUC of 0.932; sensitivity of 79.5% and specificity of 100% .

Like in other solid tumors, HNSCC must also develop direct and indirect mechanisms to induce angiogenesis. Previous studies have investigated the angiogenic expression profiles in HNSCC tumour tissues compared to normal tissue and have identified VEGF, IL-8/CXCL8, FGF-2 and HGF as key mediators of angiogenesis in HNSCC patients [34]. In addition, HGF-MET signalling pathway is known to drive the invasive phenotype of many cancers, specifically migration and metastasis in HNSCC cells [35]. Similarly, HGF levels were significantly elevated in the saliva samples collected from HNSCC patients compared to controls. This highlights that the salivary angiogenic factor changes reflect actual HNSCC tumor level, further confirming the validity of saliva testing.

Both sEGFR and sHER2 were significantly elevated in saliva samples collected from HNSCC patients compared with the saliva samples from controls. HER2 and sEGFR are both members of EGFR family, which transduce growth signals through tyrosine kinase. Overexpression of EGFR is commonly found in the tumour samples collected from HNSCC patients and it has been associated with poor prognosis and worse overall survival [36]. In addition, elevated levels of HER2 were significantly associated with short disease-free survival, overall survival and poor prognosis. HER2 receptors lack a ligand-binding domain and acts as a signal amplifier when bound to other ERBB family receptors [37]. These findings suggest that co-expression of sEGFR and sHER2 may act as prognostic biomarkers in HNSCC.

It is also known in literature that there are two models of IL-6 signalling. Classic IL-6 signalling involving a complex formation between IL-6 and membrane bound IL-6Rα whilst trans signalling involves IL-6 binding to the sIL-6Rα [38, 39]. The IL-6/IL-6Rα complex binds to ubiquitously expressed signal transducing subunit (gp130) and then complex dimerization elicits intracellular signalling [38–40]. Alternatively, the IL-6/sIL-6Rα complex acts as an agonist promoting the activity of IL-6 on cells that would otherwise be unresponsive to this cytokine due to the lack of the IL-6 receptor [39, 41]. This agonist activity of sIL-6R following IL-6 treatment was confirmed with transgenic mice and in vitro studies [42]. We have shown that sIL-6Rα levels are increased in saliva from HNSCC patients. Here we propose two mechanisms that explain the source of sIL-6R in HNSCC patients. The IL-6Rα transmembrane form is only expressed on specific cells (e.g. neutrophils, monocytes/macrophages, and some lymphocytes) [38, 40]. It is known that sIL-6Rα arises via proteolytic cleavage or alternative splicing of mRNA [43–45]. Trans signalling of sIL-6Rα derived from macrophages has shown a role in development of colorectal cancer [46]. Alternatively, mRNA expression of IL-6R and gp130 has been found in HNSCC cell lines by RT-PCR [47]. Alternate splicing may lead to the secretion of sIL-6Rα and may also play a vital role in the development of HNSCC, however further studies are warranted to establish this link.

## Conclusion

In conclusion, our findings support the use of saliva as a potential diagnostic medium to investigate angiogenic factor levels that occur in primary tumour samples. This may be an attractive way to obtain information on the angiogenic status of primary tumours if the tumours are too small to be excised via surgery. The analysis of aniogenic factors in saliva samples may provide a useful clinical alternative when tumour samples are unavailable. As an example, the majority of HPV-positive HNSCC pateints undergo chemoradiation as part of their cancer management as opposed to surgery. Future clinical trials are warranted before this panel can be implemented in a clinical setting.

## Additional file

Additional file 1: **Table S1.** Bioplex Measurements. (DOCX 17 kb)

## Abbreviation

HGF: Human Growth Factor
PECAM-1: Platelet endothelial cell adhesion molecule
sEGFR: Soluble epidermal growth factor receptor
sHER2: Soluble human epidermal growth factor receptor 2
sIL6Ra: Soluble interleukin 6 receptor antagonist
