# Supplementary material for: A pilot study to profile salivary angiogenic factors to detect head and neck cancers
Source: BMC Cancer. 2018 Jul 13;18:734. doi: 10.1186/s12885-018-4656-3 (PMC6043960; doi:10.1186/s12885-018-4656-3)
Supplement: Supplementary file 1 — Table S1. Bioplex Measurements. (DOCX 17 kb) [file 12885_2018_4656_MOESM1_ESM.docx]

Supplementary Table 1: Bioplex mesaurements

|  | Assay Performance | HNSCC Patients (n=58) | | | | | | Healthy Controls (n=38) | | | | | Diagnostic Performance | |
| --- | --- | --- | --- | --- | --- | --- | --- | --- | --- | --- | --- | --- | --- | --- |
|  | Lower Limit of Quantification (pg/mL) | Minimum | 25% Percentile | Median | 75% Percentile | Maximum | Minimum | | 25% Percentile | Median | 75% Percentile | Maximum | Area Under the Curve | P Value |
| sEGFR | 12.248 | 22.51 | 127.70 | 213.30 | 380.60 | 2119.00 | 29.18 | | 88.33 | 130.80 | 233.70 | 1654.00 | 0.65 | 0.0123 |
| FGF-basic | 5.438 | 6.78 | 37.33 | 72.01 | 114.20 | 505.50 | 7.69 | | 33.87 | 55.40 | 79.63 | 570.50 | 0.56 | 0.31 |
| Follistatin | 7.621 | 13.77 | 48.89 | 87.14 | 128.50 | 1129.00 | 13.77 | | 41.88 | 63.15 | 97.70 | 518.50 | 0.59 | 0.1579 |
| G-CSF | 0.708 | 23.08 | 103.20 | 167.90 | 299.80 | 1926.00 | 27.27 | | 77.10 | 144.60 | 272.40 | 1529.00 | 0.55 | 0.3726 |
| sHER-2/neu | 4.971 | 27.67 | 505.30 | 1092.00 | 2717.00 | 8198.00 | 24.05 | | 238.60 | 579.20 | 1110.00 | 14477.00 | 0.67 | 0.006 |
| HGF | 7.951 | 29.30 | 238.90 | 402.90 | 896.10 | 4100.00 | 30.73 | | 137.50 | 273.30 | 627.00 | 1576.00 | 0.64 | 0.0241 |
| sIL-6Rα | 0.673 | 58.89 | 485.20 | 1026.00 | 2130.00 | 10807.00 | 42.30 | | 197.20 | 506.70 | 890.70 | 3133.00 | 0.69 | 0.0013 |
| Leptin | 15.333 | 17.18 | 32.87 | 72.62 | 101.40 | 415.40 | 15.75 | | 30.60 | 54.50 | 83.88 | 521.30 | 0.56 | 0.3118 |
| Osteopontin | 11.111 | 18.70 | 46.23 | 81.65 | 116.80 | 331.50 | 17.53 | | 48.82 | 64.90 | 98.13 | 528.00 | 0.57 | 0.2471 |
| PDGF-AB/BB | 1.071 | 19.16 | 44.14 | 79.73 | 115.50 | 409.40 | 19.16 | | 40.50 | 57.50 | 94.19 | 666.00 | 0.55 | 0.4035 |
| PECAM-1 | 24.459 | 24.90 | 117.30 | 279.60 | 635.20 | 3903.00 | 25.05 | | 68.83 | 213.50 | 349.40 | 1365.00 | 0.62 | 0.0451 |
| Prolactin | 11.578 | 18.95 | 69.21 | 108.20 | 144.50 | 399.80 | 21.32 | | 67.75 | 80.65 | 125.20 | 594.80 | 0.58 | 0.2136 |
| SCF | 1.673 | 16.16 | 63.78 | 99.71 | 134.60 | 379.80 | 17.01 | | 51.63 | 76.89 | 116.10 | 536.00 | 0.60 | 0.1089 |
| sTIE-2 | 12.174 | 20.50 | 58.34 | 99.70 | 145.50 | 412.50 | 19.14 | | 50.75 | 71.00 | 105.20 | 508.00 | 0.61 | 0.0585 |
| sVEGFR-1 | 23.462 | 14.04 | 61.57 | 91.70 | 142.00 | 411.90 | 14.82 | | 56.25 | 84.70 | 118.50 | 589.50 | 0.56 | 0.3282 |
| sVEGFR-2 | 7.159 | 18.29 | 48.96 | 80.61 | 118.50 | 391.00 | 18.73 | | 37.73 | 55.50 | 88.86 | 449.30 | 0.60 | 0.05927 |
